# Magnitude of Preeclampsia and Associated Factors Among Women Attending Delivery Service in Debre Tabor Specialized Hospital

**DOI:** 10.4314/ejhs.v32i2.8

**Published:** 2022-03

**Authors:** Alemu Degu Ayele, Zemenu Alemu Tilahun

**Affiliations:** 1 Department of Midwifery, College of Health Sciences, Debre Tabor University, Debre Tabor, Ethiopia

**Keywords:** Preeclampsia, associated factors, delivery

## Abstract

**Background:**

Preeclampsia is among the leading causes of maternal and perinatal morbidity and mortality, and it continues as a global health concern. Therefore, this study was aimed to assess the magnitude of pre-eclampsia and its determinant factors among women attending delivery services in Debre Tabor Comprehensive Specialized Hospital Northwest Ethiopia.

**Methods:**

Institutional-based cross-sectional study was conducted among 261 women from January 1– 30, 2021. A systematic sampling technique was applied. Data were collected using a structured and pre-tested questionnaire. The collected data were entered using Epi-data version 4.2 and analyzed by statistical package for social science (SPSS) version 23. A significant association was declared at a p-value of < 0.05 with a 95% Confidence interval.

**Results:**

Overall 15,7% of women had preeclampsia. Age at menarche (10–15 years) (AOR=4.79; 95% CI: 2.07–15.27), unwanted pregnancy (AOR:1.29; 95% CI: 1.59–8.44), history of chronic hypertension (AOR:2.93; 95% CI: 1.00–6.20), BMI ≥ 30 Kg/m^2^ (AOR:1.79; 95% CI: 1.06–3.65), and alcohol consumption (AOR:2.12; 95% CI: 4.00–14.14) were significantly associated with preeclampsia.

**Conclusion:**

This study showed that the magnitude of preeclampsia was significantly high compared with previous national reports. Early menarche age, the status of current pregnancies, history of chronic hypertension, BMI, and alcohol consumption were significantly associated with preeclampsia. Therefore, the government and respective stakeholders should be strengthening antenatal care services to early identify and manage women with preeclampsia. Besides, health education and promotion should be strengthened regarding the maintenance of appropriate body weight and alcohol intake before pregnancy.

## Introduction

Preeclampsia is a pregnancy-related hypertensive disorder that commonly occurs after 20 weeks of gestational age. It is a syndrome characterized by the new onset of elevated blood pressure (>140/90) plus proteinuria in a previously normotensive woman (1). If preeclampsia is undetected early and left untreated, it may advance into severe life-threatening condition eclampsia characterized by the development of convulsion, which is one of the top five direct causes of maternal mortality and infant adverse outcome (1,2).

The exact etiology and pathogenesis of preeclampsia remain unclear. However, abnormal implantation of the placenta in the decidua is considered the main predisposing factor. This abnormally implanted placenta is believed to result in poor uterine and placental perfusion, which results in a state of hypoxia and increased oxidative stress, and the release of anti-angiogenic proteins into the maternal plasma along with inflammatory mediators into the maternal plasma (2).

Ethiopia is one of the African countries highly affected by maternal death which accounts for about 4.8% (14, 000) of the global share in 2017 (3). According to the Ethiopian Demographic Health Survey (EDHS) 2016 data, the maternal mortality ratio is 412 per 100,000 live births, and hypertensive disorders have a myriad role in this maternal death (4).

Worldwide, preeclampsia has remained a significant public health problem in both developed and developing countries resulting in high maternal and perinatal morbidity and mortality (5,6). However, the burden of the problem is severely high in developing countries (7), where, unlike other more prevalent causes of maternal mortality (such as hemorrhage and sepsis), medical interventions may be ineffective due to the late presentation of cases (8). In Africa and Asia, this hypertensive disorder accounts for one-tenth of all maternal mortalities, whereas onequarter of maternal deaths in Latin America have been associated with preeclampsia/ eclampsia complications (9).

The majority of deaths related to preeclampsia/eclampsia are preventable through the provision of timely identification, detection, and effective treatment of women presenting with the problem. Improving the health care system to prevent and treat women with hypertensive disorders is an essential step towards minimizing maternal and infant morbidity and mortality (10).

Globally, preeclampsia and eclampsia are responsible for ten to fifteen percent of all maternal deaths (11, 12), whereas in Ethiopia 85% of maternal deaths were associated with the five leading direct causes of maternal death namely, hemorrhage, obstructed labor, preeclampsia/eclampsia, unsafe abortion, and sepsis. Of these five leading causes of maternal deaths, preeclampsia/eclampsia accounts for 11% (13,14).

The prevalence rate of preeclampsia was 3.3 per 100 person-years in Australia (15), and 5.2 per 1,000 person-years in Yorkshire (16). The incidence rate of preeclampsia in developing countries varies from 1.8–16.7% (17–19). In Ethiopia it also varies from 1.2% (20) to 19.1% (21).

The government of Ethiopia has implemented multi-pronged approaches to reducing maternal and newborn morbidity and mortality by improving access to and strengthening facility-based maternal and newborn services. However, maternal morbidity and mortality due to hypertensive disorder are still on an alarming trend (13).

Even though preeclampsia is a leading cause of maternal morbidity and mortality during pregnancy, labor and delivery little is known about the current prevalence of preeclampsia, its associated factors among women attending delivery services in Ethiopia and particularly in the study areas. Therefore, this study was intended to assess the prevalence of preeclampsia and its associated factors among women attending labor and delivery in the Debre Tabor Comprehensive Specialized Hospital. The result of this study will be important for improving the quality of life and survival status of mothers and newborn babies and the sustainable economic growth of the country at large.

## Methods

**Study design and setting**: An institution-based cross-sectional study was conducted in Debre Tabor Comprehensive Specialized Hospital from January 1–30, 2021. Debre Tabor Comprehensive Specialized Hospital is found in Debre Tabor Town, South Gondar district of Amhara Regional state which is about 665 kilometers far from Addis Ababa (the capital city of Ethiopia) in a Northwest direction and 103 kilometers from Bahir Dar. The Hospital delivers community health care including maternal and child health services. All women attending delivery service in the hospital during the study periods were the study population. Women who have given written informed consent to participate in the study and attend delivery service in the hospital were included while; women with known hypertension and renal disease were excluded from the study. Also, participants who were critically ill and unable to communicate during the study period were excluded.

**Sample size and Sampling procedure**: A total of 261 study participants were included by using single population proportion formula with the assumption of a proportion of preeclampsia was 19.1% (21), the margin of error (d) 5%, and by adding a 10 percent non-response rate. A systematic sampling technique was employed to recruit the study participants. Based on the hospital report on average 372 pregnant women were gave birth each month. We calculated the K^th^ interval, which was 1.47∼2. Of the first two participants, one woman was randomly selected by using the lottery method. Afterward, we selected and included the study participant every K value interval until the desired number of participants were obtained.

**Data collection procedure quality control**: The data were collected via face-to-face interview techniques using a structured and pre-tested questionnaire. The questionnaire was first developed in English then converted to the local Amharic language for clarity and back to English for consistency by two separate language expert individuals who speak both English and Amharic fluently. The questionnaire was developed after an intensive review of relevant kinds of literature. A pre-test of the questionnaire was done on 5% of the total participants (13 women) in Addis Zemen primary Hospital near to the study setting and necessary modifications such as; wording, logical sequence, and skip patterns were made immediately. The data were collected by two diploma midwives and supervised by one trained BSc Midwife. Data collectors and supervisor were trained for one day regarding the objective of the study, items of the questionnaires, confidentiality, and informed consent before the actual data collection. The completeness and consistency of data were cross-checked, cleaned, and compiled daily by supervisor and principal investigator.

**Data processing and analysis**: The collected data were coded, cleaned, and entered into Epi-Data version 4.2 then transferred to SPSS version 23 for analysis. Descriptive statistics including tables and percentages were used to explain the data. Binary and multivariable logistic regression analyses were carried out. Variables that showed association in binary logistic regression analysis with P-value less than 0.20 were entered into a multivariable logistic regression analysis model for further analysis. Finally, a significant association was declared at a p < 0.05 and adjusted odds ratio (AOR) with 95% CI.

**Ethical consideration**: Ethical clearance was obtained from the institutional Ethics Review Committee of College of Health Sciences, Debre Tabor University. Besides this, a support letter was issued from Debre Tabor Hospital. Written informed consent was gained from each study participant after explaining the purpose of the study. To keep the privacy and assure confidentiality of the respondents, any personal identifiers were excluded.

## Results

**Socio-demographic characteristics**: In this study, a total of 261 participants were included with a response rate of 100%. The participants' age ranged from 18–44 years with a mean age of 27.59 (SD ± 6.39) years. The majority of participants, 243 (93.1%) were Amhara by ethnicity and 221 (84.7%) were Orthodox Christian followers. Of the total participants, 242 (93.7%) were married and 107 (41.0%) were housewives (Table 1).

**Table 1:** Socio-demographic characteristics of study participants in Debre Tabor Comprehensive Specialized Hospital Northwest Ethiopia, January 1–30, 2021(N=261)

Characteristics	Frequency	Percent
**Age**		
15–24	68	26.1
25–34	126	48.3
35–49	67	25.6
Mean (SD), yr.		27.59 (SD ± 6.39)
**Residency**		
Urban	117	44.5
Rural	144	55.5
**Ethnicity**		
Amhara	243	93.1
Others*	18	6.9
**Religion**		
Orthodox	221	84.7
Muslim	30	11.5
Others**	10	3.8
**Educational status**		
No formal	56	21.5
education	51	19.5
Primary	87	33.3
Secondary	67	25.7
Tertiary		
**Occupational** **status**		
Housewife	107	41.0
Government employee	64	24.5
Private business	80	30.7
Others***	10	3.8
**Marital status**		
Married	242	93.7
Others****	19	7.3

Note:

* Oromo, Tigray, Gurage

** protestant, catholic

*** employed at private sector, student and job finder

**Obstetric and reproductive health characteristics**: Of the total respondents, one hundred sixty (61.3%) and one hundred one (38.7%) were primigravids and multigravidas respectively. Regarding ANC follow-up two hundred twenty-one (84.7%) women had at least one ANC follow-up. About two hundred fortythree (93.1%) of women had no family history of PE. Concerning the current status of pregnancies near to three froths (71.3 %) of the current pregnancies were wanted and planned (Table 2).

**Table 2:** Obstetric and reproductive health characteristics of study participants in Debre Tabor Comprehensive Specialized Hospital, Northwest Ethiopia, January 1–30, 2021(N=261)

Characteristics	Frequency	Percent
**Gravidity**		
Primi	160	61.3
Multi	101	38.7
**Age at menarche**		
≤ 15 years	43	16.5
>15 years	218	83.5
**ANC follow up**		
Yes	221	84.7
No	40	15.3
**Change partner**		
Yes	56	21.5
No	205	78.5
**History of abortion**		
Yes	32	12.7
No	229	87.7
**Family history of PE**		
Yes	18	6.9
No	243	93.1
**Have PE currently**		
Yes	41	15.7
No	220	84.3
**Diastolic BP(N=41)**		
90–110	28	68.3
> 110	13	31.7
**Condition of pregnancy**		
Wanted	186	71.3
Unwanted	46	17.6
Mistimed	29	11.1

**Medical and lifestyle characteristics**: From the total sample of 261, 14.2% of participants had gestational diabetes mellitus in their current pregnancies and 13.4% had a history of chronic hypertension. Near two-thirds of the participants (68.2%) had taken alcohol at least during holly days. About one hundred eighty-one (69.4%) of respondents had a normal BMI of 18–24.9 Kg/m^2^. About two hundred three (78.8%) and one hundred seventy-one (65.5%) had taken vegetables and fruits respectively (Table 3).

**Table 3:** Medical and Life style related characteristics of the study participants in Debre Tabor Comprehensive Specialized Hospital, Northwest Ethiopia, January 1–30, 2021(N=261)

Characteristics	Frequency	Percent
**History of GDM**		
Yes	37	14.2
No	224	85.8
**Family history of DM**		
Yes	22	8.4
No	239	91.6
**History of renal disease**		
Yes	30	11.5
No	231	88.5
**Family history of HTN**		
Yes	36	13,8
No	225	86.2
**History of chronic HTN**		
Yes	36	13.8
No	225	86.2
**Smoking habit**		
Yes	13	5.0
No	248	95.0
**Alcohol intake**		
Yes	178	68.2
No	83	31.8
**Coffee intake**		
Yes	173	66.3
No	88	33.7
**BMI**		
< 18	21	8.0
18–24.9	181	69.4
25–29.9	30	11.5
≥ 30	29	11.1
**Vegetable's intake**		
Yes	203	77.8
No	58	22.2
**Fruit intake**		
Yes	171	65.5
No	90	34.5

**Magnitude of preeclampsia**: According to this study finding, 41 (15.7%) with 95% CI (11.3–19.9) of the participants had preeclampsia. Of these women 28 (68.3%) and 13 (31.7%) had a diastolic blood pressure measurement of 90–110 mmHg and ≥ 110 mmHg respectively. Of the total 41 cases, 35 (85.4%) cases were diagnosed during labor and delivery whereas the remainin (14.6%) were diagnosed during their ANC follow-up. All preeclamptic women were taken anticonvulsant medication (magnesium sulfate) and antihypertensive (methyldopa) and none of them were develop eclampsia.

**Factors associated with preeclampsia**: In binary logistic regression analysis age at menarche, the status of current pregnancy, change partner before the current pregnancy, family history of preeclampsia, history of chronic hypertension, smoking habit, BMI, alcohol intake were significantly associated with the development of preeclampsia.

As can be depicted from multivariable backward stepwise likely hood logistic regression analysis, age at menarche (10–15 years), the status of current pregnancy, history of chronic hypertension, BMI ≥ 30 Kg/m^2^, and alcohol intake were remained significantly associated with preeclampsia.

Women whose age at menarche ranged from 10–15 years were nearly five times more likely to develop preeclampsia (AOR: 4.79; 95% CI: 2.07–15.27) than women whose age at menarche were greater than 15 years. Women whose pregnancies were unwanted and unplanned were 1.29 times higher odds of developing preeclampsia (AOR: 1.29; 95% CI: 1.59–8.44) as compared to those women whose pregnancies were wanted and planned. Similarly, study participants who had a history of chronic hypertension were nearly three times more likely to develop preeclampsia (AOR: 2.93; 95% CI: 1.00–6.20) than their counterparts. Once more, respondents who had a BMI of ≥ 30 Kg/m^2^ were nearly two times the odds of developing preeclampsia (AOR: 1.79; 95% CI:1.06–3.65) as compared to those women who had a BMI of < 18Kg/m^2^. Lastly, women who had consuming alcohol intake were 2.12 times higher odds of developing preeclampsia (AOR: 2.12; 95 % CI: 4.00–14.14) as compared to their counterparts (Table 4).

**Table 4:** Bivariable and multivariable analysis of factors affecting preeclampsia among mothers following labor and delivery in Debre Tabor Comprehensive Specialized Hospital, Northwest Ethiopia, January 1–30, 2021(N=261)

Variables	Preeclampsia		COR (95%CI)	AOR (95%CI)	*P-value*
	Yes (%)	No (%)			
Age in years					
15–24	10 (14.7)	58(85.3)	1	1	
25–34	15(11.9)	111(88.1)	1.276(0.539–3.018)	1.439(0.207–10.02)	0.713
≥35	16 (23.9)	51(76.1)	0.550(0.229–1.318)	3.343(0.267–8.456)	0.349
Age at menarche					
10–15 years	20(46.5)	23 (53.5)	8.157(3.856–17.258)	4.796(2.070–15.270)	0.009*
15–20 years	21(9.6)	197(90.4)	1	1	
Gravidity					
Primi	28(17.5)	132(82.5)	0.696(0.342–1.418)	1.987(0.897–8.896)	0.385
Multi	13(12.9)	88(87.1)	1	1	
Status of pregnancy					
Wanted	8(4.3)	178 (95.7)	1	1	
Unwanted	22(47.8)	24 (52.2)	0.049(0.020–0.122))	1.295(1.597–8.442)	0.045*
Mistimed	11(37.9)	18(62.1)	0.074(0.392–0.206)	0.954(0.294–4.824)	0.932
Change partner					
Yes	25 (44.6)	31(55.4)	0.105(0.050–0.219)	2.876(0.654–5.213)	0.893
No	7.8(51.1)	189(92.2)	1	1	
Family history of PE					
Yes	13 (72.2)	5(27.8)	0.050(0.017–0.151)	1.064(0.787–3.098)	0.076
No	28(11.5)	215(88.5)	1	1	
History of chronic HPN					
Yes	11(30.6)	25(69.4)	0.35(0.156–0.783)	2.932(1.005–6.204)	0.000*
No	30(13.3)	195(86.7)			
Smoking habit					
Yes	7(53.8)	6(46.2)	0.136(0.043–0.430)	1.876(0.021–5.472)	0.092
No	34(13.7)	214(86.3)	1	1	
BMI					
<18 Kg/m^2^	6(28.6)	15(71.4)	1	1	
18–24.9 Kg/m^2^	9(5.0)	172(95,0)	7.644(2.397–24.384)	4.654(0.387–12.536)	0.987
25–29.9 Kg/m^2^	9(30.0)	21(70.0)	0.933(0.274–3.184)	6.925(0.759–10.183)	0.086
≥ 30 Kg/m^2^	17(58.6)	12(41.4)	0.282(0.085–0.938)	1.796(1.068–3.650)	0.005*
Alcohol intake					
Yes	34(19.1)	144(80.9)	0.39(0.165–0.922)	2.121(4.001–14.148)	0.001*
No	7(8.4)	76(91.6)	1	1	

Note:

* p-value < 0.05 considered as statistically significant

## Discussion

According to this study, the magnitude of preeclampsia among women attending labor and delivery service in Debre Tabor comprehensive specialized hospital was 15.7%. This finding was in line with a study conducted in Finland (13.9%) (22) Nigeria (16%) (23), Mettu (12.4%) (24), Arba Minch (18.25%) (25), Gondar (16.8%) (26), and Jijiga (19.1%) (21).

However, our finding was higher than a study done in India (7.8%) (27), Iran (9.8%) (28), Tanzania (3.3%) (29), Ghana (7.5%) (30), Cameron (4.44%) (31), Mizan Tepi (7.9%) (32), Halaba (9.9%) (33), Dessie (8.4%) (34). The discrepancies might be explained due to differences in the geographical locations, culture and lifestyle, study period, study design, and healthcare-seeking behaviors of pregnant women. Besides, the dissimilarity might be due to the government's full engagement to introducing focused ANC and special emphasis on pregnant women, such as exempted maternal care services which raises the healthcare-seeking behavior of pregnant women and delivery at health facilities which improves case detections. Once more, this could be due to a real increase in the number of cases of preeclampsia, which would necessitate more such a large-scale study.

Our study finding noted that PE was significantly correlated with young age at menarche (10–15 years). The finding was consistent with a study conducted in Karnataka (35). The possible explanation might be due to an increased risk of experiencing cardiovascular events that have been associated with early menarche (10–15 years). In addition, preeclampsia can theoretically be facilitated by increased fatty tissue accumulation accompanied by early menarche (36).

In the current finding, women whose pregnancies were unwanted and unplanned were at increased risk of developing preeclampsia. To the best of our knowledge, this is the first study that identified the status of pregnancy as a predictor of preeclampsia. The probable explanation might be those women with an unintended and unplanned pregnancy has no healthcare-seeking behavior, including ANC follow-up that allows them to obtain information on danger signs of pregnancy as a result of PE (headache, blurring of vision, epigastric pain), as well as its preventive modalities and timely intervention before it worsens. In the meantime, women with unwanted and unplanned pregnancies may experience stressful life events and mental issues that may adversely affect the women's overall health status and the incidence of preeclampsia might be exacerbated by this stress full lifestyle.

Our study finding also identified that pre-existing chronic hypertension was positively associated with the development of preeclampsia. Similarly, different studies have shown that women with pre-existing chronic hypertension have a higher risk of developing preeclampsia (23, 25,37). The potential reason could be women with a previous history of chronic hypertension might have an increased risk of coronary heart disease, stroke, heart failure, and kidney disease which were easily associated with preeclampsia.

According to the current study finding, women who had a BMI of ≥ 30Kg/m^2^ were at increased risk of developing preeclampsia. This finding was in agreement with different study findings which reported that overweight and obesity were an important predictors of preeclampsia (29, 38,39). Even though the precise pathophysiology of the predisposition of obesity and overweight to preeclampsia is unclear, it is postulated that hyperinsulinism, insulin resistance, and maternal systemic inflammation are strongly linked to obesity and overweight. These abnormalities may lead to endothelial dysfunction, hypertension, proteinuria, preeclampsia, and multi-organ damage which are frequently happening in PE (40).

Lastly, as stated elsewhere, alcohol consumption was highly predisposed to the development of preeclampsia (25, 26). The probable reason may be that alcohol use might be affecting the renal function and systemic blood vessels that may expose the individual to secondary hypertension and potentially lead to preeclampsia.

This study has its drawbacks. First, there might be recall bias concerning some variables such as time of menarche. Secondly, biochemical tests for the exclusion of women with other chronic medical conditions have not been performed. Last but not least, the cross-sectional nature of the study didn't allow us to approve whether non-preeclamptic women remained negative until discharged to home or not.

In conclusion, the magnitude of preeclampsia was significantly high as compared with a previous national reports which was 6.82%. Early menarche age, the status of current pregnancies, history of chronic hypertension, BMI, and alcohol consumption were significantly associated with preeclampsia. Therefore, the government and respective stakeholders should be strengthening antenatal care services to early identify and manage women with preeclampsia. Besides, health education and promotion should be strengthened regarding the maintenance of appropriate body weight and alcohol intake before pregnancy. Lastly, this study suggests that further studies should be conducted to assess lifestyle and dietary factors using analytical study designs.

## References

[1] American College of Obstetricians and Gynecologists (2013). Hypertension in Pregnancy, Task force on hypertension in pregnancy. Obstet Gynecol.

[2] Green P (2014). Update in the Diagnosis and Management of Hypertensive Disorders in Pregnancy.

[3] WHO U, UNFPA, World Bank Group and the United Nations Population Division (2019). Trends in Maternal Mortality 2000 to 2017.

[4] Central Statistical Agency (CSA) [Ethiopia] and ICF (2016). Ethiopia Demographic and Health Survey 2016: Key Indicators Report.

[5] Dolea C, AbouZahr C (2003). Global burden of hypertensive disorders of pregnancy in the year 2000. GBD 2000 Working Paper.

[6] McClure EM, Saleem S, Pasha O, Goldenberg RL (2009). Stillbirth in developing countries: a review of causes, risk factors and prevention strategies. The journal of maternal-fetal & neonatal medicine.

[7] Igberase GO, Ebeigbe PN (2006). Eclampsia: tenyears of experience in a rural tertiary hospital in the Niger delta, Nigeria. Journal of obstetrics and gynaecology.

[8] Onakewhor JU, Gharoro EP (2008). Changing trends in maternal mortality in a developing country. Nigerian Journal of Clinical Practice.

[9] Al-Jameila N (2014). A brief overview of preeclampsia. J Clin Med Res.

[10] World health organization (2011). Recommendations for prevention and treatment of pre-eclampsia and eclampsia.

[11] Khan KS, Wojdyla D, Say L, Gülmezoglu AM, Van Look PF (2006). WHO analysis of causes of maternal death: a systematic review. The lancet.

[12] Duley L (2009). The global impact of pre-eclampsia and eclampsia. Semin Perinatol.

[13] Federal Democratic Republic of Ethiopia Ministry of Health (2010). Health sector development Programme IV 2010/11 – 2014/15.

[14] Federal Democratic Republic of Ethiopia Ministry of Health (2013). Maternal death surveillance and response (MDSR) technical guideline.

[15] Thornton C, Dahlen H, Korda A, Hennessy A (2013). The incidence of preeclampsia and eclampsia and associated maternal mortality in Australia from population-linked datasets: 2000–2008. Am J Obstet Gynecol.

[16] Tuffnell D, Jankowicz D, Lindow S (2005). Group YOCC. Outcomes of severe preeclampsia/eclampsia in Yorkshire 1999/2003. BJOG.

[17] Fekadu GA, Kassa GM, Berhe AK, Muche AA, Katiso NA (2018). The effect of antenatal care on use of institutional delivery service and postnatal care in Ethiopia: a systematic review and meta-analysis. BMC Health Serv Res.

[18] Ngwenya S (2017). Severe preeclampsia and eclampsia: incidence, complications, and perinatal outcomes at a low-resource setting, Mpilo Central Hospital, Bulawayo, Zimbabwe. Int J Womens Health.

[19] Osungbade KO, Ige OK (2011). Public health perspectives of preeclampsia in developing countries: implication for health system strengthening. J Pregnancy.

[20] Gaym A, Bailey P, pearson L, Admasu K, Gebrehiwot Y, Ethiopian National Em ONCAT (2011). Disease burden due to preeclampsia/eclampsia and the Ethiopian health system's response. International Journal of Gynaecology and Obstetrics: the official organ of the International Federation of Gynaecology and Obstetrics.

[21] Mekonen L, Shiferaw Z, Wubshet Ea, Haile S (2018). Pregnancy Induced Hypertention and Associated Factors among Pregnant Women in Karamara Hospital, Jijiga, Eastern Ethiopia, 2015. Journal of Pregnancy and Child Health.

[22] Kaaja R, Kinnunen T, Luoto R (2005). Regional differences in the prevalence of pre-eclampsia in relation to the risk factors for coronary artery disease in women in Finland. Eur Heart J.

[23] Guerrier G, Oluyide B, Keramarou M, Grais RF (2013). Factors associated with severe preeclampsia and eclampsia in Jahun, Nigeria. Int J Women's Health.

[24] Belay AS, Wudad T (2019). Prevalence and associated factors of pre-eclampsia among pregnant women attending anti-natal care at Mettu Karl referal hospital, Ethiopia: cross-sectional study. Clinical hypertension.

[25] Shegaze M, Markos Y, Estifaons W, Taye I, Gemeda E (2016). Magnitude and Associated Factors of Preeclampsia Among Pregnant Women who Attend Antenatal Care Service in Public Health Institutions in Arba Minch Town, Southern Ethiopia, 2016. Gynecol Obstet (Sunnyvale).

[26] Walle TA, Azagew AW (2019). Hypertensive disorder of pregnancy prevalence and associated factors among pregnant women attending ante natal care at Gondar town health Institutions, North West Ethiopia 2017. Pregnancy hypertension.

[27] Sajith M, Vandana NV, Modi A, Sumariya R, Pawar A (2014). Incidence of pregnancy induced hypertension and prescription pattern of antihypertensive drugs in pregnancy. International Journal of Pharma Sciences and Research.

[28] Khosravi S, Dabiran S, Lotfi M, Asnavandy M (2014). Study of the Prevalence of Hypertension and Complications of Hypertensive Disorders in Pregnancy. Open Journal of Preventive Medicine.

[29] Mrema (2018). The association between pre pregnancy body mass index and risk of preeclampsia: a registry based study from Tanzania. BMC Pregnancy and Childbirth.

[30] Adu-Bonsaffoh K, Obed SA, Seffah JD (2014). Maternal outcomes of hypertensive disorders in pregnancy at Kore Bu Teaching Hospital, Ghana. Int J Gynaecol Obstet.

[31] Ngowa JDK, Kasia JM, Alim J (2015). Maternal and perinatal complications of sever preeclampsia in the referal hospitals in Yaounde, Cameroon. Open J Obst Gynecol.

[32] Gudeta Tesfaye Abera, Regassa Tilahun Mekonnen (2018). Pregnancy Induced Hypertension and Its Associated Factors among Women Attending Delivery Service at Mizan-Tepi University Teaching Hospital, Tepi and Gebretsadikshawo Hospitals, Southwest, Ethiopia. Ethiop J Health Sci.

[33] Andarge RB, Anshebo AA, Halil HM, Kebede BA, Abdo RA (2020). Prevalence and Associated Factors of Pre-eclampsia among Pregnant Women at Antenatal Booking in the Halaba Kullito General Hospital, Southern Ethiopia. J Women's Health Care.

[34] Tessema GA, Tekeste A, Ayele TA (2015). Preeclampsia and associated factors among pregnant women attending antenatal care in Dessie referral hospital, Northeast Ethiopia: a hospital-based study. BMC Pregnancy Childbirth.

[35] Osungbade KO, Ige OK (2011). Public Health Perspectives of Preeclampsia in Developing Countries: Implication for Health System Strengthening. Journal of Pregnancy.

[36] Latha K, Judie A (2014). Unveiling The Precedence of Risk Factors for Pre-Eclampsia: for Suitable Surveillance and Prophylaxis. IJSRInternational Journal Of Scientific Research.

[37] Lecarpentier E, Tsatsaris V, Goffinet F, Cabrol D, Sibai B (2013). Risk factors of superimposed preeclampsia in women with essential chronic hypertension treated before pregnancy. Plos One.

[38] Kahsay HB, Gashe FE, Ayele WM (2018). Risk factors for hypertensive disorders of pregnancy among mothers in Tigray region, Ethiopia: matched case-control study. BMC Pregnancy Childbirth.

[39] Mohammed E, Agero G, Ali E (2017). Preeclampsia risk factors among pregnant women attending in four Public Health Facilities of Addis Ababa City. Ethiopian Journal of Reproductive Health (EJRH).

[40] Lopez-Jaramillo P, Barajas J, Rueda-Quijano SM, Lopez-Lopez C, Felix C (2018). Obesity and Preeclampsia: Common Pathophysiological Mechanisms. Front Physiol.

